# Modified negative pressure wound therapy as an adjunct to antibiotics in the treatment of orthopaedic infected metalwork

**DOI:** 10.1007/s00590-021-03135-5

**Published:** 2021-10-02

**Authors:** Selina Summers, Natasha Faye Daniels, Azeem Thahir, Matija Krkovic

**Affiliations:** 1 School of Clinical Medicine, University of Cambridge, Addenbrooke’s Hospital, Cambridge, CB2 0SP UK; 2 Department of Trauma and Orthopaedics, Addenbrookes Major Trauma Unit, Cambridge University Hospitals, Cambridge, UK

**Keywords:** Saline solution, Vacuum-assisted closure, Negative pressure wound therapy, Infected orthopaedic metalwork, Hardware salvage

## Abstract

**Purpose:**

Infected orthopaedic metalwork is challenging to treat. Negative pressure wound therapy (NPWT) with irrigation is an emerging therapy for infected wounds as an adjunct to antibiotic therapy. The senior author had devised a modified technique to augment its efficacy, utilising high-flow rate irrigation and skin closure over the standard NPWT dressing. This novel technique was originally evaluated in a different centre and produced 100% success in metalwork retention. The present study is a reproducibility test of the same technique.

**Methods:**

A retrospective review was performed on 24 patients with infected orthopaedic metalwork, including 3 upper limb and 21 lower limb cases, for outcomes relating to implant retention and infection resolution. Patients underwent a modified NPWT technique as an adjunct to antibiotic therapy and surgical debridement. Detailed medical and microbiology information were obtained from the patient records.

**Results:**

23 of 24 (96%) patients had successful metalwork retainment with healed wounds and resolution of infection, allowing fracture union. 27 infective organisms were identified in this cohort, and the antibiotic regimens for each patient are provided. The average follow-up was 663 days. No adverse effects were observed.

**Conclusion:**

This series supports the modified NPWT technique as a safe, reliable and effective adjunct therapy to resolve metalwork infection. The same results have been reproduced as the previous cohort in a different centre.

**Supplementary Information:**

The online version contains supplementary material available at 10.1007/s00590-021-03135-5.

## Introduction

Metalwork infection is a troublesome complication for orthopaedic surgeons, typically following hardware implantation or surgical site infection. These can be particularly hard to treat; surgical removal of infected metalwork is undesirable as it is associated with intensive treatment, higher costs and instability [1].

Negative pressure wound therapy (NPWT) is an increasingly popular method to promote wound healing in orthopaedic surgery, with Vacuum Assisted Closure® (VAC)® being the most common variant used [2]. The local subatmospheric pressure and fluid drainage are proposed to aid the formation of granulation tissue, increase blood flow and reduce oedema to improve wound healing [3]. Modified NPWT techniques have been extensively used in wounds of mixed aetiologies such as laparotomy wounds, extremity ulcers and spinal wounds [4, 5]. Some of these modified techniques are now being explored in orthopaedic wounds as an adjunct to antibiotic therapy and surgical debridement in implant salvage procedures.

Our paper reflects on a modified technique in which continuous saline irrigation at −125 mmHg continuous pressure was used along with direct wound closure in layers over the NPWT for patients with infected metalwork. This enabled the metalwork to remain in situ whilst simultaneously treating the infection, reducing the likelihood of leakage and loss of pressure that a standard technique would confer. The aim is to adequately suppress the infection to allow time for fracture union with the retained metalwork. The wound closure over the NPWT dressing has the benefits of preventing skin contracture and contributes to an infection barrier.

This is a repeat study of Norris et al. (2013) [6] with the same technique as the original, carried out by the same surgeon. Variations of modified NPWT are common in the literature, but few are carefully repeated to assess the validity of their results. We test our modified technique’s reproducibility which is crucial for its validation, to ensure that any differences in the rate of metalwork retainment and infection resolution results from changes in pathology and not from the variability of the technique. Aside from differences in time and location, it was impossible to duplicate every condition of the 2013 study [6], such as microbiology advice. Some of the limitations of the 2013 study were also addressed, such as longer follow-up time.

## Methods

We conducted a retrospective study on 42 patients treated by a single orthopaedic surgeon (MK) who had the modified NPWT over a 7 year duration (2013–2020) from a single centre. Inclusion criteria included the presence of metalwork in situ and sufficient follow-up data available. Patients were excluded if there was no metalwork in situ, if metalwork was removed prior to the use of NPWT or if they had arthroplasty or hemiarthroplasty (due to different treatment protocols). Metalwork was retained if it was clinical stable (for example, screws were solidly fixed in the bone and plate was firmly against the bone); otherwise, it was removed and replaced. Each patient was on an antibiotic regimen to target the causative organism, and thus, due to the heterogeneity of the patients, the antibiotic regimens were variable and based on microbiologist advice from the bone infection team.

Three authors reviewed the patients’ electronic and paper medical records available in the form of surgical data, infectious disease data, discharge summaries and post-operative follow-up letters. Data pertaining to the demographics of the patients, smoking status, comorbidities, length of follow-up and whether the infection was early (< 8 weeks after operation) or late (> 8 weeks) was gathered. In regards to the defect itself, information was gathered on bone loss, the presence of a soft tissue defect and the date and cause of injury. For the VAC® dressing, the indication (e.g. wound dehiscence), the number of changes and the time period between changes were noted. Microbiological data in the form of the causative organism and antibiotic regimen and duration were gathered. The long-term outcomes after infection resolution and any wound specific complications were included.

**Fig. 1:**
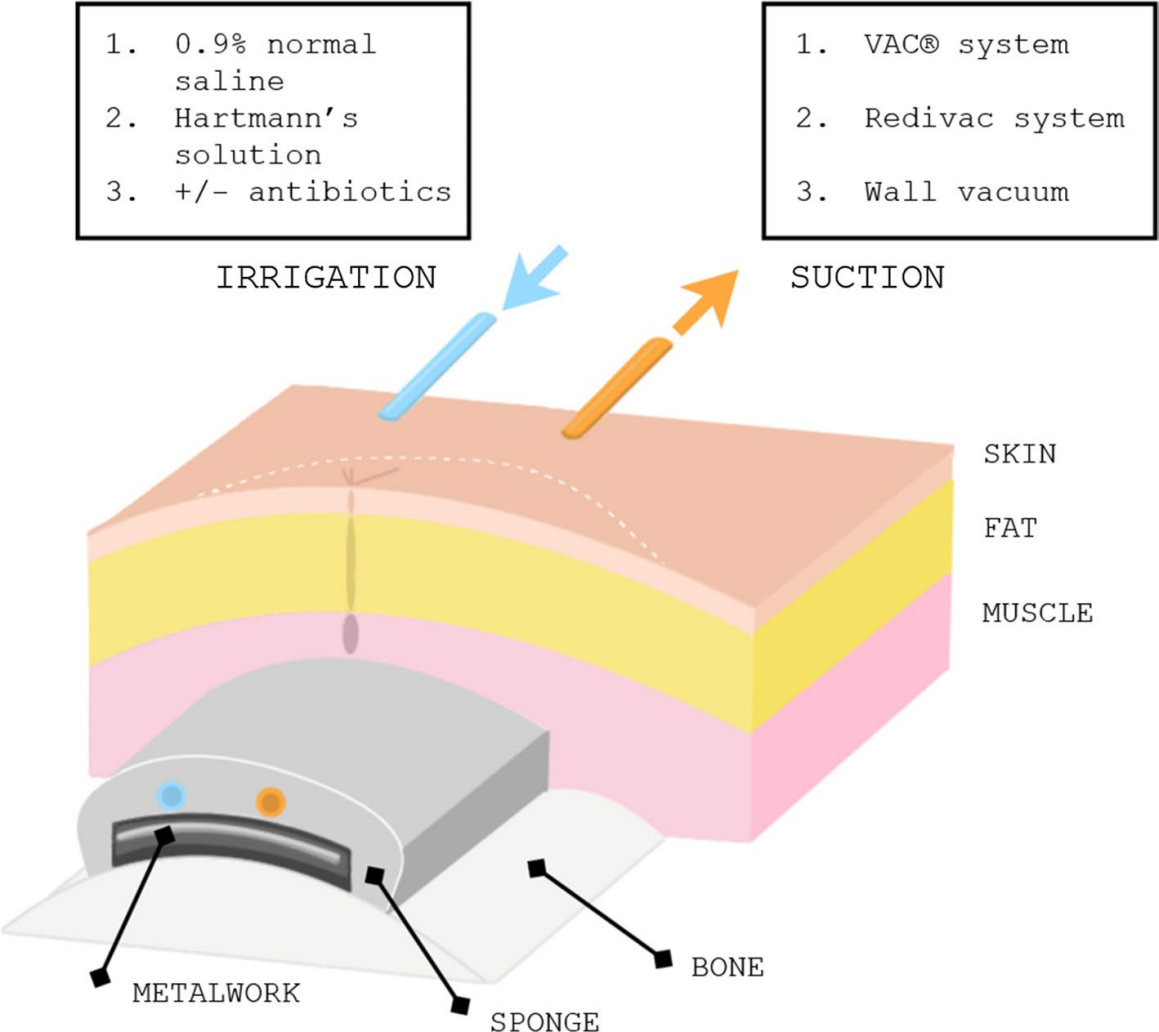
Illustration of the modified NPWT irrigation with skin closure over sponge. Irrigation and suction arrangements are detailed, connected to a VAC machine with the fluid set to run in at a rate of 2L per 24 h. VAC, vacuum-assisted closure

Each patient underwent initial debridement of the infected wound, with metalwork left in situ. A VAC dressing was then applied directly onto the metalwork with a hydrophilic small pore polyvinyl foam lining (KCI, Kidlington, UK) (Fig. 1). This was used instead of the wide pore black polyurethane foam (GranuFoam™; KCI, Kidlington, UK) enabling a longer period in which the foam can be left in situ. A 16G Redivac drain (Biomet, Bridgend, UK) was then connected to suction at −125 mmHg continuous pressure, and a 10G Redivac drain was connected to saline for irrigation. The initial drip rate was approximately 2000 mL saline per 24 h in the first 48 h, which was reduced to 1000 mL per 24 h depending on sponge size.

**Fig. 2:**
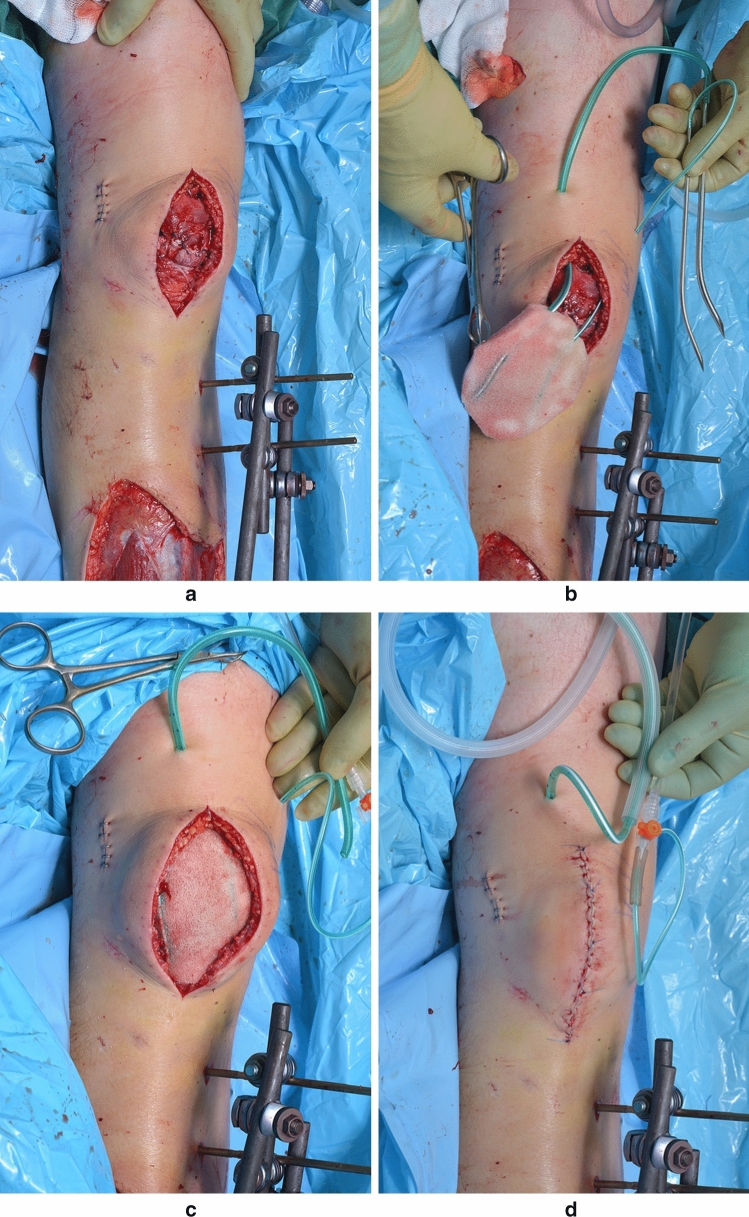
A series of photographs demonstrating the modified NPWT technique from **a** initial debridement to **b** the fitting of two Redivac drains led out through the skin and into the sponge. **c** Next, the foam was placed in situ into the wound and **d** the wound was closed in layers over the NPWT dressing after which the suction tube and fluid administration tubing was connected to check if the system was functioning well

Wound closure was then performed over the hydrophilic foam in layers, using interrupted absorbable synthetic braided sutures around the deep fascia, and continuous synthetic non-absorbable nylon sutures for skin closure (Fig. 2). The patients returned to theatre for a second debridement after ~ 6 days, with the VAC dressing changed and modified to accommodate any wound shrinkage. At this stage, the definitive debridement was done as the growing new granulation tissues helped with distinguishing between a healthy base with granulation tissue and a necrotic base without granulation tissue growing. This was repeated in 6–8 days. If there was any doubt of infection clearance, the dressing was exchanged and kept in place for another week. In each change, the sponge put in would be smaller to minimise the dead space as it would also allow an easier wound closure. The VAC dressing was then removed one week after last change and the wound closed over a Redivac drain for 72 h.

Patients were followed up, and the presence of infection was assessed with clinical examination and laboratory findings including erythrocyte sedimentation rate (ESR), C-reactive protein (CRP) and white cell count (WCC). The absence of swelling, redness and pain with the laboratory parameters within normal ranges constitute no active infection.

Ethical approval for the study was not required as this was a retrospective study without any patient intervention. Signed informed consent was obtained from patients prior to the procedure as well as for using their information without patient identifying data. All sensitive data were anonymised with patient-identifying elements removed.

## Results

A total of 42 patients had undergone modified NPWT for any indication (summarised in Table 1). A subgroup of 24 patients (15 male and 9 female; average age 50.4 years, range 21–85 years) had infected metalwork in situ. Of this subgroup, 19 were early-onset infections, while 5 were of late-onset. These patients were separated into upper limb and lower limb groups (Tables 2, 3). The average duration of NPWT was 19 days, and the average number of visits to theatre for NPWT change or removal was 2.5 (mode: 2).

**Table 1:** Summary of indications for modified NPWT

Reason for therapy	Number of patients
Infections with metalwork in situ	24
Osteomyelitis with no metalwork involved	10
Soft tissue cases with no metalwork involved	6
Metalwork removed prior to the use of NPWT	1
Infected arthroplasty/hemiarthroplasty	1

All patients undergoing negative pressure wound therapy (NPWT) in our centre between 2013 and 2020 with the indications

**Table 2:** Results of upper limb group (*n* = 3)

Patient number	Patient age	Early/late infection	Procedure	Duration of NWPT irrigation (days)	Number of NWPT changes	Long-term outcome
1	29	Early	ORIF clavicle	23	2	Wound healed, no infection
2	27	Late	Arthroscopic latarjet procedure shoulder	34	5	Wound healed, no infection
3	31	Early	ORIF clavicle	14	2	Wound healed, no infection (later had hook plate removed)

Patient, wound and treatment characteristics, and long-term outcome. *ORIF* open reduction, internal fixation

**Table 3:** Results of lower limb group (*n* = 21)

Patient number	Patient age	Early/late infection	Procedure	Duration of NWPT irrigation (days)	Number of NWPT changes	Long-term outcome
4	54	Late	Hip DHS	27	4	Wound healed, no infection
5	58	Early	ORIF calcaneum	15	2	Wound healed, no infection
6	34	Early	ORIF talus	15	2	Wound healed, no infection, talonavicular joint arthritis
7	48	Early	ORIF ankle	17	3	Wound healed, no infection
8	38	Early	ORIF ankle	15	1	Wound healed, no infection
9	59	Early	Osteotomy post tibial nail	20	3	Wound healed, no infection
10	53	Early	ORIF patella	23	3	Wound healed, no infection (later had metalwork removal to allow bone healing)
11	44	Early	Ex-fix knee followed by multiligament reconstruction medial tibial plateau	14	2	Wound healed, no infection; recurrent cellulitis around the area, very slow recovery (later had some metalwork removed with 1 washer and 1 screw fragment left in situ)
12	57	Late	Ex-fix tibia	10	1	Wound healed, no infection (later had all metalwork removed and TSF in situ)
13	28	Late	Ex-fix followed by IM nail to tibia with 1st stage Masquelet cement spacer insertion	17	3	Wound healed, no infection
14	32	Early	ORIF pilon fracture	7	1	Wound healed, no infection, mild swelling
15	40	Early	Left tibial revision ORIF of plateau fracture	21	3	Wound healed, no infection
16	78	Late	Revision total knee replacement	11	2	Wound healed, no infection, no active sinus
17	57	Early	Adjustment of external fixator femur	26	3	Above knee amputation and prosthetic leg; wound healed, no infection
18	21	Early	Left femoral valgus osteotomy and greater trochanteric shortening osteotomy, left periacetabular pelvic osteotomy	14	2	Wound healed, no infection (later had removal of blade plate, 6 screws and 2 washers)
19	85	Early	Closed reduction insertion DHS femur	27	3	Persistent infection; deceased
20	66	Early	ORIF femur	28	2	Wound healed, no infection
21	58	Early	ORIF tibia	14	2	Wound healed, no infection
22	56	Early	ORIF femur and bone transport	21	2	Wound healed, no infection (later had part of LRS nail removed after NPWT therapy, followed by one episode of late recurrent infection resolved with a second NPWT)
23	73	Early	ORIF NOF fracture and DHS	28	3	Wound healed, no infection
24	83	Early	THR periprosthetic fracture ORIF	23	3	Wound healed, no infection

Patient, wound and treatment characteristics, and long-term outcome. *DHS* dynamic hip screw; *ORIF* open reduction, internal fixation; *Ex-fix* external fixation; *TSF* Taylor spatial frame; *IM* intramedullary; *LRS* limb reconstruction system; *NOF* neck of femur; *THR* total hip replacement

Overall, 23 of 24 (96%) patients had successful retention of metalwork with resolution of the acute infection from the initial presentation which allowed the fractures to unite or the ongoing orthopaedic procedures to continue. One patient with multiple comorbidities had persistent infection even after completing NPWT therapy and died months after from reasons unrelated to her infected metalwork.

After fracture union or procedure completion, the remaining 23 patients were followed up (mean 663 days, range 17–2045 days) for wound status and late signs of infection. Three of these patients (patients 11, 17, 22) displayed late signs of infection despite resolution of the initial acute infection and had metalwork removal for unrelated reasons. Treatment of the early infection allowed patient 11 to achieve fracture union; however, he later had metalwork removal for knee fusion, with an episode of mild leg cellulitis over the previous surgical site. Patient 17 completed his bone lengthening procedure after treatment with the modified NPWT, however he later had amputation of the same leg due to non-correctable varus deformity of the foot. Patient 22 successfully completed his bone transport procedure, however he later had a recurrent infection and nonunion of the distal femur. His nail broke and was replaced, followed by a second modified NPWT for 32 days with 3 NPWT changes. He had healed wounds and no further recurrence of infection and the time to the last follow-up was 690 days.

Similarly, four other patients (patients 3, 10, 12, 18) had all or some metalwork removed after completion of the modified NPWT treatment for reasons relating to other parts of their management, with no late signs of infection. To date, 16 patients with the original metalwork in situ have complete resolution of infection with no recurrence. Overall, 20 of 23 (87%) patients had no recurrent infection after modified NPWT. Wound-related complications were rare and those identified were chronic inactive sinus, mild swelling and recurrent infection.

The most common infective organisms were MSSA, Enterobacter cloacae, Propionibacterium acnes and Staphylococcus epidermidis (Table 4). Overall, there were 27 infective organism categories identified amongst the 24 patients, and their detailed antibiotic regimens according to the bone infection team advice are outlined in the Supplementary Material. 23 of 24 patients received OPAT treatment.

**Table 4:** Microbiology summary of common infections

Causative organism	Organism incidence	Antibiotics used
MSSA	5	Ceftriaxone, ciprofloxacin, clarithromycin, co-amoxiclav, ertapenem, flucloxacillin, fusidic acid, meropenem, rifampicin, tazocin, teicoplanin, vancomycin
Enterobacter cloacae	5	Ciprofloxacin, co-amoxiclav, ertapenem, flucloxacillin, gentamicin, meropenem, rifampicin, tazocin, teicoplanin, vancomycin
Propionibacterium acnes	2	Amoxicillin, ceftriaxone, ciprofloxacin, co-amoxiclav, flucloxacillin, rifampicin, teicoplanin
Staphylococcus epidermidis	2	Ciprofloxacin, co-amoxiclav, ertapenem, meropenem, rifampicin, teicoplanin, vancomycin

Causative organisms ranked in order of highest incidence with antibiotics used listed. MSSA, methicillin-susceptible *Staphylococcus aureus*

Other reported causative organisms with incidence of one include Citrobacter koseri, coagulase-negative Staphylococcus, Coliform bacilli, Corynebacterium amycolatum, Diphtheroids, Enterobacter asburiae, Enterobacter kobei, Enterobacter sp., Enterococcus faecium, ESBL Enterobacter cloacae, *Escherichia coli*, Finegoldia magna, Group B streptococcus, Klebsiella pneumoniae, Klebsiella variicola, Micrococcus luteus, MRSA, Neisseria, Proteus sp., skin flora, Staphylococcus hominis, Staphylococcus lugdunensis, Streptococcus sp

## Discussion

The orthopaedic experience in treating infected metalwork has been marginally explored with promising results [6, 7]. Our primary endpoint is the retention of metalwork and resolution of active infection, allowing fracture union; our present cohort has shown a 96% success rate, reflecting the 100% success rate in the previous cohort (*n* = 16) [6]. Reviews of the literature have shown largely 100% success rates from case series using NPWT instillation focussing on implant salvage in extremity wounds (*n* = 4) [8], spinal wounds (*n* = 3) [9] and infected left ventricular assist devices (*n* = 3) [10]. A recent retrospective review (*n* = 28) showed 89% successful retention or replacement of implants of mixed locations (spinal, sternal and extremity), and specifically 61% success in original implant retention [7]. This is similar to a previous clinical observational study which demonstrated 86% (*n* = 22) orthopaedic implant retention in acute infection and 80% (*n* = 10) in chronic infection [11]. The results from our study and Norris et al. [6] suggest an advantage to our particular modified NPWT technique in consistently producing implant salvage.

The infective organism is important, as it guides antibiotic treatment and prognosis. In our cohort, no single organism consistently caused recurrent infection. Differentiation between easy- and hard-to-treat pathogens is impractical as it depends on antimicrobial resistance, properties of metalwork material surface and the quality of debridement surgery. Differentiation between early- and late-onset infections reveals that metalwork retention is suitable in both cases [6], despite many advocating for early cases only.

Defining metalwork infection treatment success is a topic of much debate. Although not directly applicable, one international consensus study [12] established a multidimensional definition for periprosthetic joint infection, including infection eradication, no subsequent surgical intervention for infection and no mortality related to the infection. We remain cautious about claiming infection eradication, as we cannot monitor for biofilm activity indefinitely. However, the aim of this technique is to produce clinically adequate suppression of infection whilst allowing metalwork retainment for bone healing. Seven patients received subsequent surgical removal of metalwork, none of which were owed to infection.

Our modified technique should be re-evaluated in the light of contemporary study findings. Firstly, the effectiveness of continuous irrigation in NPWT over standard NPWT remains disputed. Recent randomised controlled trials (RCTs) have directly compared standard NPWT with antiseptic or saline irrigation in complex foot infections and found no significant difference in outcomes [13, 14]. Conversely, another RCT reported improved granulation tissue, reduced wound surface area and reduced bacterial load in extremity ulcers in NPWT with saline instillation over standard NPWT [4]. We highlight the paucity of controlled clinical studies for the use of NPWT irrigation in orthopaedic patients. To our best knowledge, there is no RCT for standard NPWT and NPWT with copious saline irrigation.

Secondly, while the ideal irrigation fluid remains a subject of debate, we believe the fluid’s main objective within our technique is to prolong sponge patency, subsequently avoiding further surgery and secondary infections. Both saline and antiseptic fluids have demonstrated a significant reduction in bacterial load over control in a porcine model [15], and similar effectiveness in NPWT instillation [16]. Additionally, saline has demonstrated a greater proportion of surgical wound closure, better wound healing, reduced hospital stay, reduced length of therapy and greater cost-effectiveness [4, 5, 17] and is recommended as first-line by the international consensus guidelines [18]. The guidelines also recommended an antiseptic solution if hardware is involved for the initial 24–48 h, followed by saline instillation, which avoids the cytotoxic effect that accompanies long-term use. From our results, we believe that saline is sufficient.

Thirdly, our flow rate follows a two-step approach with an initial rate (2L/day, ~ 83 mL/h) that is twice as high as the maintenance rate (1 L/day, ~ 42 mL/h). This differs from the more conventional choice of a constant rate of 15–30 mL/h in the literature. Similarly, another study [5] adopted an even greater rate of 3L/day of normal saline with VAC combined with closed suction irrigation system (CSIS). They reported a shortened average wound healing time of 17 days, a shortened hospital stay of 33 days and no recurrent infection. Present studies have disputed the relevance of flow rate. An animal study compared flow rates of ~ 15 mL/h and ~ 40 mL/h and found no difference in outcome [15]. However, we believe that the rates commonly used in the literature to simulate ‘high’ flow rate are often insufficient to detect a difference, and a more rigorous study is needed to detect the effect of copious irrigation.

Finally, testing the reproducibility of any novel techniques is an important step as is the present study. While numerous variations of NPWT have been independently tested in the literature [7–11] to produce implant salvage, rigorous testing of one technique is rare. We reproduced similar results as the previous cohort in a different location [6], which reconfirms the data. We consider this a high degree of agreement to the previous data based on its continued success in suppressing the infection. The other strengths of our study are consistent technique and a long follow-up period of almost two years on average.

### Limitations

Our study is limited with a small sample size, a heterogeneous range of conditions and a single performing surgeon. The design is observational and retrospective in nature with no controls and randomisation, where the decision to perform the modified NPWT is purely clinical. There may have been selection bias due to identification of a previously successful cohort [9], but this is minimised with the broad inclusion criteria. There is a heterogeneous mix of causative organisms and antibiotics used. These results are generated from a single centre so may not be generalisable due to different wound care protocols and microbiology advice. However, antimicrobial plans were culture specific and made by infection specialists with appropriate reviews, in line with the BOAST guidelines. [19]

## Conclusion

The present study provides more contemporary information regarding the modified NPWT technique in managing infected metalwork. The modified NPWT with copious saline irrigation is safe and reliable with over 96% success rate in metalwork retainment and resolution of acute infection in extremity cases. This improved technique is a valuable adjunct to conventional antibiotic therapy in orthopaedic patients with retained infected metalwork.

## Supplementary Information

Below is the link to the electronic supplementary material.

Supplementary file1 (PDF 81 kb)

## Data Availability

All data are available in the main text.
