# Supplementary material for: Modified negative pressure wound therapy as an adjunct to antibiotics in the treatment of orthopaedic infected metalwork
Source: Eur J Orthop Surg Traumatol. 2021 Oct 2;32(8):1561–8. doi: 10.1007/s00590-021-03135-5 (PMC9587958; doi:10.1007/s00590-021-03135-5)
Supplement: Supplementary file 1 — Supplementary file1 (PDF 81 kb) [file 590_2021_3135_MOESM1_ESM.pdf]

SUPPLEMENTARY INFORMATION

Table Detailed microbiology of infections

**Patient number**	**Causative organism(s)**	**Antibiotics used, average dose (duration in weeks (w) and days (d))**
1	Coagulase-negative Staphylococcus	Ceftriaxone 2g (3w6d)<br>Teicoplanin 600mg (1w)
2	Propionibacterium acnes, Diphtheroids	Teicoplanin 600mg (8w3d)<br>Ciprofloxacin 750mg (8w3d)<br>Rifampicin 300mg (1d)
3	Propionibacterium acnes, Neisseria	Flucloxacillin 500mg (3w)<br>Co-amoxiclav 625mg (1w1d)<br>Ceftriaxone 2g (1w)<br>Rifampicin 300mg (8w6d)<br>Ciprofloxacin 750mg (9w6d)<br>Amoxicillin 1g (9w6d)
4	Citrobacter koseri	Meropenem 500mg (3w1d)<br>Ciprofloxacin 750mg (4w1d)
5	Enterobacter cloacae	Meropenem 50mg (2w1d)<br>Ertapenem 1g (3w6d)<br>Ciprofloxacin 700mg (1d)
6	Enterobacter cloacae	Meropenem 500mg (2w1d)<br>Ertapenem 1g (6w)<br>Ciprofloxacin 750mg (1w3d)
7	MSSA	Clarithromycin 500mg (1w1d)<br>Vancomycin 1g (1w2d)<br>Teicoplanin 600mg (6w3d)<br>Rifampicin 300mg (9w5d)<br>Ciprofloxacin 750mg (2w)
8	MSSA	Meropenem 500mg (3d)<br>Rifampicin 300mg (7w5d)<br>Ertapenem 1g (1w3d)<br>Vancomycin 1g (3d)<br>Tazocin 4.5g (3d)<br>Flucloxacillin 1g (1w2d)
9	MSSA	Vancomycin 1g (3d)<br>Meropenem 500mg (3d)<br>Flucloxacillin 2g (2w3d)<br>Rifampicin 300mg (4w1d)<br>Ceftriaxone 2g (1d)<br>Fusidic acid 500mg (1w4d)
10	MSSA	Flucloxacillin 2g (3w)<br>Ceftriaxone 2g (3w)<br>Rifampicin 300mg (2w6d)

11	MSSA	Co-amoxiclav 1.2g (4d)<br>Tazocin 4.5g (2w3d)<br>Vancomycin 1g (2w3d)<br>Ceftriaxone 2g (1w)<br>Rifampicin 300mg (1w)
12	Streptococcus	Vancomycin 1g (6d)<br>Benzylpenicillin 1.2g (1w1d)<br>Ceftriaxone 2g (11w6d)<br>Flucloxacillin 1g (1d)<br>Tazocin 4.5g (3d)<br>Amoxicillin 500mg (1d)
13	Group B Streptococcus	Co-amoxiclav 1.2g (3w)<br>Ceftriaxone 2g (4w4d)
14	Skin flora, Enterobacter cloacae	Tazocin 4.5g (1w)<br>Vancomycin 1500mg (1w)<br>Flucloxacillin 1g (STAT)<br>Gentamicin 120mg (STAT)<br>Ciprofloxacin 750mg (12w)<br>Teicoplanin 1g (5w)
15	Finegoldia magna, Staphylococcus lugdunensis	Flucloxacillin 1g (1d)<br>Vancomycin 1.75g (2w)<br>Rifampicin 300mg (1w5d)<br>Ciprofloxacin 750mg (1w)<br>Daptomycin 600mg (2w)<br>Metronidazole 400mg (4w)
16	Staphylococcus capitis, Staphylococcus epidermidis	Vancomycin 1g (1w)<br>Tazocin 4.5g (1w)<br>Rifampicin 300mg (3d)<br>Teicoplanin 800mg (4d)
17	Corynebacterium striatum	Co-amoxiclav 1.2g (1d)<br>Meropenem 1g (1w)<br>Tazocin 4.5g (4d)<br>Vancomycin 1.25g (3w2d)<br>Daptomycin 700mg (1w6d)<br>Rifampicin 300mg (1w6d)
18	Finegoldia magna, Staphylococcus haemolyticus	Teicoplanin 1g (1w2d)<br>Ciprofloxacin 750mg (1w3d)<br>Daptomycin 1g (6w5d)<br>Meropenem 1g (4w3d)<br>Fluconazole 100mg (6d)
19	Enterococcus faecium, Micrococcus luteus, Staphylococcus epidermidis, Enterobacter sp, Enterobacter cloacae, Klebsiella pneumoniae, Enterobacter asburiae, Enterobacter kobei	Co-amoxiclav 1.2g (1w3d)<br>Vancomycin 1g (12w)<br>Ciprofloxacin 500mg (3d)<br>Meropenem 1g (3w6d)

20	MRSA, Klebsiella variicola	Co-amoxiclav 1.2g (5d)<br>Gentamicin 120mg (STAT)<br>Meropenem 2g (4d)<br>Vancomycin 1.5g (1d)<br>Tazocin 4.5g (2w)
21	Coliform bacilli, ESBL<br>Enterobacter cloacae	Vancomycin 2.5g (5d)<br>Flucloxacillin 1g (STAT)<br>Ciprofloxacin 750mg (4w2d)<br>Ertapenem 1g (5w)
22	Enterobacter cloacae,<br>Staphylococcus epidermidis,<br>Staphylococcus hominis	Rifampicin 300mg (2w)<br>Ciprofloxacin 400mg (4d)<br>Ertapenem 1g (2w1d)<br>Teicoplanin 1.2g (6w4d)
23	Proteus sp, Escherichia coli,<br>Corynebacterium amycolatum	Vancomycin 1.5g (2w)<br>Gentamicin 120mg (STAT)<br>Ciprofloxacin 750mg (5w5d)<br>Tazocin 4.5g (1w)<br>Meropenem 1g (8w4d)
24	None identified	Flucloxacillin 1g (STAT)<br>Gentamicin 200mg (STAT)<br>Co-amoxiclav 1.2g (2w4d)<br>Tazocin 4.5g (3d)<br>Vancomycin 1g (2d)<br>Meropenem 1g (3w)<br>Ciprofloxacin 750mg (11w4d)<br>Ertapenem 1g (3d)<br>Metronidazole 400mg (1w)<br>Daptomycin 600mg (8w4d)

MSSA, methicillin-sensitive Staphylococcus aureus; MRSA, methicillin-resistant Staphylococcus aureus; ESBL, extended-spectrum β-lactamase
